# Simplified engineering geomorphic unit-based seismic site characterization of the detailed area plan of Dhaka city, Bangladesh

**DOI:** 10.1038/s41598-023-37628-6

**Published:** 2023-07-10

**Authors:** Md Shakhawat Hossain, Muneyoshi Numada, Momtahina Mitu, Kishor Timsina, Chaitaniya Krisna, Md. Zillur Rahman, A. S. M. Maksud Kamal, Kimiro Meguro

**Affiliations:** 1 Institute of Industrial Science, The University of Tokyo, Tokyo, Japan; 2 Department of Disaster Science and Climate Resilience, University of Dhaka, Dhaka, Bangladesh; 3 Department of Coastal Studies and Disaster Management, University of Barisal, Barisal, Bangladesh; 4 Department of Civil and Infrastructure Engineering, Asian Institute of Technology, Pathum Thani, Thailand

**Keywords:** Natural hazards, Civil engineering

## Abstract

Severe failure of improperly designed and poorly constructed structures may occur due to the amplified and prolonged ground motion during an earthquake, and hence prediction of the ground motion characteristics at the soil surface is crucial. In this study, based on the prepared simplified engineering geomorphic map, we performed a one-dimensional (1D) nonlinear site response analysis for seismic site characterization of the recently proposed Detailed Area Plan (DAP) area of Dhaka City, the Capital of Bangladesh. The engineering geomorphic unit-based map was prepared from image analysis and verified with the collected borehole data and surface geology map. The study area was classified into three major geomorphic units and seven sub-units subject to the subsurface soil profiles. Nine earthquake time histories, seven from the PEER NGA WEST2 data set and two synthetics, and seven identified subsurface soil profiles were used for nonlinear site response analysis, along with the BNBC 2020 uniform hazard spectrum as the target spectrum. For the selected earthquake ground motions, the near-surface soil response of the DAP area showed de-amplification of acceleration in the short period and amplification of acceleration in the long period. The amplified long-period acceleration could cause severe damage in inappropriately designed and poorly constructed long-period structures. The outcome of this study could be used to prepare a seismic risk-sensitive land use plan for the future development of the DAP of Dhaka City.

## Introduction

Bangladesh, a low-lying deltaic country, is one of the most vulnerable countries to natural and anthropogenic hazards due to its geographical location, topography, monsoon climate, and dense population. It experienced over 200 disasters between 1980 to 2018^1^. Floods, cyclones, river bank erosion, storm surge, drought, salinity intrusion, fire, and earthquake are common hazards for this country. Even though floods and cyclones regularly occur in Bangladesh, the Himalayan system in the north and the Arakan subduction-collision system in the east are the two major tectonic systems that can produce large-magnitude earthquakes in and around Bangladesh^2^. The history of earthquakes in the recent past and the present rate of tectonic plate movement suggest that Bangladesh and its surrounding area are seismically active^3^. However, seismic vulnerability mainly depends on strong ground motion and the quality of the built environment. The strong ground motion characteristics of engineering bedrock (shear wave velocity (Vs) > 760 m/s) can be significantly amplified or de-amplified due to local site conditions, i.e., subsurface soil conditions. The earthquakes of 1995 Kobe, 1989 Loma Prieta, 1985 Mexico, 1964 Alaska, and 1964 Niigata earthquake witnessed the damage associated with effects of site conditions^4,5^. Therefore, analyzing the site response to know the local site effects for performance-based seismic design is a prerequisite to making a resilient society.

Site response analysis aims to estimate the earthquake ground motions by determining how the local soil properties of any site influence the expected rock motions^6^. The most simplified site response analysis procedure is based on the site amplification factor calculated using the average Vs of top 30 m soil (Vs30) based site amplification factor^7,8^. However, this simplified procedure is unsuitable when the engineering bedrock depth is more than 30 m^9^. Therefore, to get an acceptable ground response, using the dynamic properties of the soil column along with bedrock ground motion as input is preferred^10–13^. Researchers performed 1D site response analysis with linear, equivalent-linear, and nonlinear models and obtained the acceptable surface ground motion^14–16^. Since soil behavior is nonlinear, some adjustments to the linear technique are required for estimating ground response. The equivalent-linear approach is an improved site response strategy with better results than the linear approach. A computer-based program termed "SHAKE" for equivalent-linear approximation demonstrated the actual hysteresis behavior of cyclic loading of soil^17^. However, when soil shear forces are more than 10^−5^ kN/m^2^, the soil takes on a nonlinear behavior^18^. The nonlinear model is more specific than the equivalent-linear method in representing the hysteresis stress–strain reaction of soil. Frequency and time domain analyses are used to determine the site response's nonlinear effects^19^. Site response analysis can be carried out using various computer programs; for example, SHAKE2000^20^, SHAKE91^21^, and SHAKE^22^ are for 1D equivalent-linear, and DEEPSOIL^23^, OpenSees^24^, and SUMDES^25^ are for nonlinear site response analysis.

However, a few studies have been conducted in Bangladesh to estimate surface ground motion using site response analysis. For example, Rahman et al.^9^ performed site response analysis based on Vs30-based site coefficient, linear, equivalent-linear, and nonlinear approaches at ten locations of Dhaka city corporation area^9^. In addition, Ansary and Jahan^26^ performed an almost similar site response analysis for a solar power plant site in the northern part of Bangladesh^26^. However, detailed and extensive nonlinear site response is essential as soft soil is present in Dhaka city's newly proposed DAP area. In addition, no published research considered simplified engineering geomorphic units of DAP of Dhaka city for seismic site characterization by nonlinear site response analysis. To our knowledge, this study is the first to combine both approaches for seismic site characterization in this specific area of Dhaka city.

Dhaka City is Bangladesh's administrative, political, and economic capital, with nearly 20–30% of the country's Gross Domestic Product (GDP) from Dhaka City. The city ranked as one of the highest densely populated cities in the world in 2022, and the built-up area changed from 1989 to 2020 by around 92%^27^. The vigorous expansion of Dhaka city has recently created a large built environment over soft soil. The seismic vulnerability of Dhaka city is very high due to its geographic location, population density, unplanned urbanization, and non-engineered construction practice^28^. If any earthquake occurs with a magnitude greater than 7.0, it is approximated that more than 72,326 buildings will be damaged^29^.

Consequently, to reduce vulnerability and make a beautiful habitable city, the Government of Bangladesh (GoB) planned for detailed area planning in Dhaka City^30,31^. Different geotechnical, environmental, network analysis, etc. analyses are needed for detailed area planning. The Sendai Framework for Disaster Risk Reduction (DRR) (2015–2030) sets seven vibrant targets and four priorities for action to prevent new and reduce existing disaster risks. One of the priorities of the Sendai Framework is understanding disaster risk. Therefore, a detailed consideration of geomorphic unit-wise seismic site characterization is needed to minimize future seismic risk and create a resilient built environment.

The graphical representation of geomorphic features for an engineering application is known as engineering geomorphic mapping^32^. Geomorphic features can be identified, categorized, quantified, and visualized for site characterization using mapping. As part of the pre-development planning to reduce risk after development, geomorphic mapping is helpful for site-specific projects to detect and describe existing or potential geotechnical and hydro-technical hazards^32^. Kamal and Midorikawa^33^ prepared a GIS-based geomorphological map with landfill sites only for the Dhaka city area. Further, they performed surface soil response analysis using boreholes and horizontal-to-vertical (H/V) spectral ratio (microtremor data) based on predominant periods and corresponding amplification factors^33^. Later, Rahman et al.^34,35^ prepared a simplified geomorphic map of Dhaka city based on Vs30 and the liquefaction hazard map. However, the geomorphic map proposed by Kamal and Midorikawa^33^ and Rahman et al.^34,35^ was only for the Dhaka City Corporation area (around 320 km^2^); the present study covered the newly proposed DAP area of Dhaka City (around 1500 km^2^). In addition, we have further simplified the geomorphic map by merging similar units as it will be easy to implement for practitioners.

Currently, Dhaka City is considered as one of the mostly seismic vulnerable cities; there is a scope and need for a seismic risk-sensitive land use plan for the proposed DAP area of Dhaka City to reduce seismic risk. Therefore, the objective of this study is to: (i) prepare a simplified engineering geomorphic unit map of the DAP of Dhaka City, (ii) delineate the engineering properties of each unit, and (iii) perform extensive nonlinear site response analysis of each unit considering the similar response spectra to the Bangladesh National Building Code (BNBC) 2020 for maximum credible earthquake (MCE) and SB type soil condition (Vs. 360 to 800 m/s).

The outcome of this study could be helpful in seismic risk-sensitive land use planning (RSLUP) for the future development of the DAP area to reduce seismic risk.

## Geology and seismotectonics of the study area

**Figure 1:**
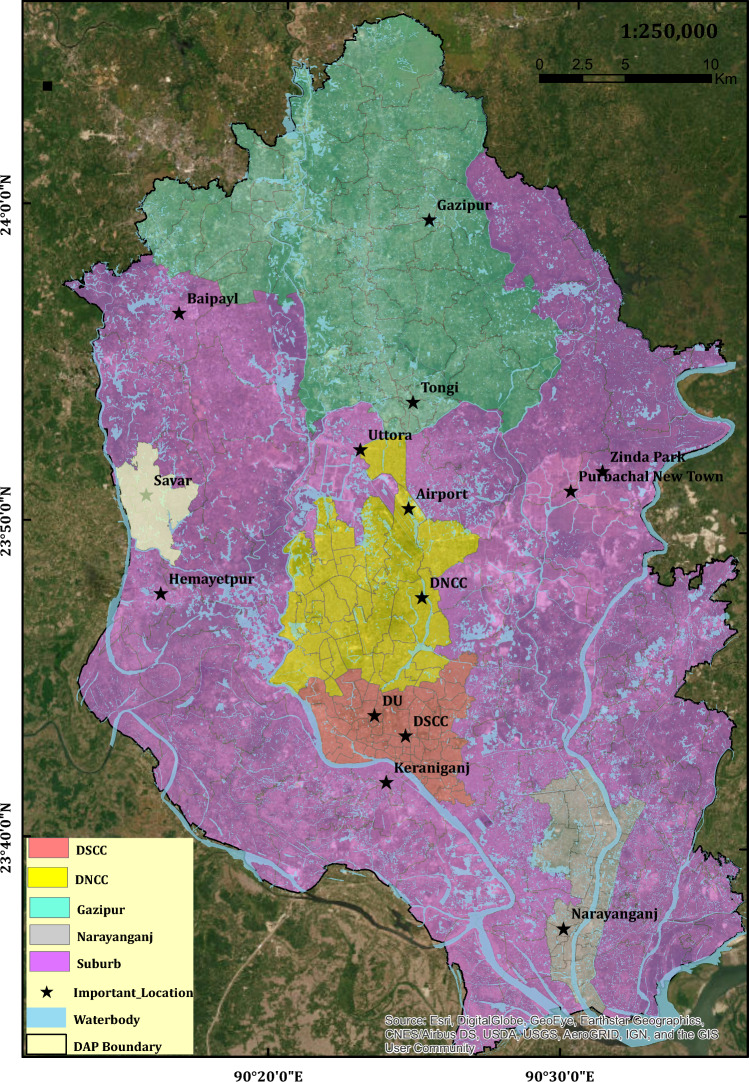
Location Map of the Study Area (DAP are of Dhaka City) [DSCC: Dhaka South City Corporation, DNCC: Dhaka North City Corporation, DU: Dhaka University]. The map was produced using ArcGIS.

The area of DAP of Dhaka City is approx. 1528.44 sq. km; DAP is included from Gazipur to Narayanganj, covering Savar, Old Dhaka, and Keraniganj (Fig. 1). According to Bangladesh's seismic zonation map, the DAP area is in Zone II and has a seismic zone coefficient of 0.15^36^. Uplifted Pleistocene Madhupur terraces and Holocene floodplains^33,35^ are the two major surface geological units of the DAP area of Dhaka. The Pleistocene terrace consists of a yellowish brown medium stiff to very stiff clayey silt (Madhupur clay) and is underlain by medium dense to dense silty sand (Dupitila sand). However, the Holocene alluvium, or floodplains, are grey, very soft to medium stiff clay/ silty clay, and very loose to medium dense silt/ clayey silt with silty sand/sand beneath^34,37^.

DAP of Dhaka City is almost at the Bengal basin's center. Bengal basin, formed by the Ganges–Brahmaputra-Meghna River system, is one of the world's largest and most active deltas. It is unique because of its colossal sediment thickness (approximately 22 km^38^). The Himalayan Ranges between the Indian Plate and Eurasian Plate, as well as the Bengal Basin in the eastern part of the Indian Plate, were both produced by the northward collision of the Indian Plate with the Eurasian Plate^39–42^. The Indian Plate bounds the Bengal basin to the west, the Eurasian Plate to the north, the Burmese Plate to the east, and the Bay of Bengal to the south and includes Bangladesh and parts of West Bengal, Tripura, and Assam of Indian states. The Himalayan system (product of the Indian-Eurasian plate) in the north and the Arakan subduction-collision system (product of the Indian-Burma plate) are the two major tectonic systems that have the potential to produce large earthquakes in the Bengal Basin area (Supplementary Fig. S1)^43^.

The large and destructive earthquakes in Bangladesh, northern and northeastern India, Nepal, Bhutan, and Myanmar were caused by two significant active tectonic belts: the Indian-Eurasian plate and the Indian-Burma plate^44^. The 1897 Great Indian Earthquake significantly damaged buildings in Dhaka. Supplementary Fig. S1 shows the earthquakes (with a magnitude of Mw ≥ 5) located in and around Bangladesh between 1908 and 2015. The tectonic belt of the Himalayan system is expected to experience large earthquakes between M 7.5 and M 8.5 as the Indian plate moves northward at a rate of 4 cm/year and northeastward at a rate of 6 cm/year^45^. The Dauki Fault, which is thought to have caused the 1897 Great Indian Earthquake^46^, was activated three times in the last thousand years, according to recent paleo-seismological research^47–49^. As a result, the rate of plate movement and regular occurrence of large-magnitude earthquakes along this plate boundary suggests that Bangladesh, Nepal, Bhutan, Myanmar, and the northern and northeastern parts of India, are seismically active regions^43^.

The Chittagong-Tripura fold belt has a noticeably different landscape due to the N-S trending anticlines, which resulted from the collision of the Indian and the Burmese plates, resting on the décollement megathrust of the arc-trench system of the Indian plate and the Burma Arc^50^. This zone is a consequence of the ongoing deformation along the subduction zone. In addition, the Neogene deformation system of the Chittagong-Tripura fold belt has minor seismicity indicating a state of locked condition of the plates^44^.

## Method

This study prepared a simplified engineering geomorphic map of the Dhaka DAP area. Then, over two hundred boreholes with various lab test results and downhole seismic test data are collected to verify the simplified engineering geomorphic unit and delineate unit-wise engineering properties. Later, nine strong ground motion data are selected to execute site response analysis. Supplementary Fig. S2 shows the methodological framework adopted for this study. Further details of each component are explained in the subsequent sub-sections.

### Simplified engineering geomorphic map

To reduce earthquake risk, an engineering geomorphic map is essential for zoning any area. It is a land classification map based on the geomorphic unit, which helps to know the soil type variation on the surface. We have used both boreholes’ data and satellite images to prepare the engineering geomorphic map. The Landsat 5 image of 1988 and the Landsat 8 image for 2020 from USGS Earth Explorer are used for this research and are corrected using radiometric and atmospheric correction. Using the image from 1988, we digitized the basic geographic unit through visual interpretation (using natural and false colors). We then verified the geomorphic unit map from boreholes data and surface geology data from GSB (Geological Survey of Bangladesh). Later, NDWI (Normalized Difference Water Index) detected water bodies and landfills for both images of 1988 and 2020. We extract the water bodies from images by NDWI, give values 0 to 1, and then analyze the changes of water bodies in the images. From the studied images, landfill areas and water bodies are detected for the present time. Then these two units are added to the previously prepared map.

### Engineering soil properties

Primary and secondary data have been collected to delineate subsurface engineering properties. Primary data includes Dhaka University Master Plan and Development of Open Space Management System to Response Scenario Earthquake in Dhaka Metropolitan Area projects data as some authors were directly involved in acquiring, processing, and interpreting Standard Penetration Test (SPT) borehole, geotechnical lab test, and downhole seismic test.

The secondary data are collected from the Geological Survey of Bangladesh (GSB), Creative Soil Investigations, Prosoil, Comprehensive Disaster Management Programme (CDMP). All data contain information about soil geotechnical properties, SPT N value, lithological properties, grain size, liquid limit, plastic limit, shear wave velocity (Vs), cohesion, friction angle, etc.

We have identified three major types of soil profiles from the collected borehole data considering the geomorphic units. They are Pleistocene terrace (overconsolidated clay or silty clay or clayey silt (OC)), Holocene alluvium (HA), and Landfill (LAN). Further, OC is divided into OC 1, OC 2, and OC 3 based on the subsurface soil layer. Similarly, HA and LAN are divided as HA 1, HA 2, and LAN-OC LAN-HA, respectively. LAN-OC and LAN-HA mean LAN underlay by OC and HA, respectively.

### Site response analysis

#### Shear wave velocity of the study area

**Figure 2:**
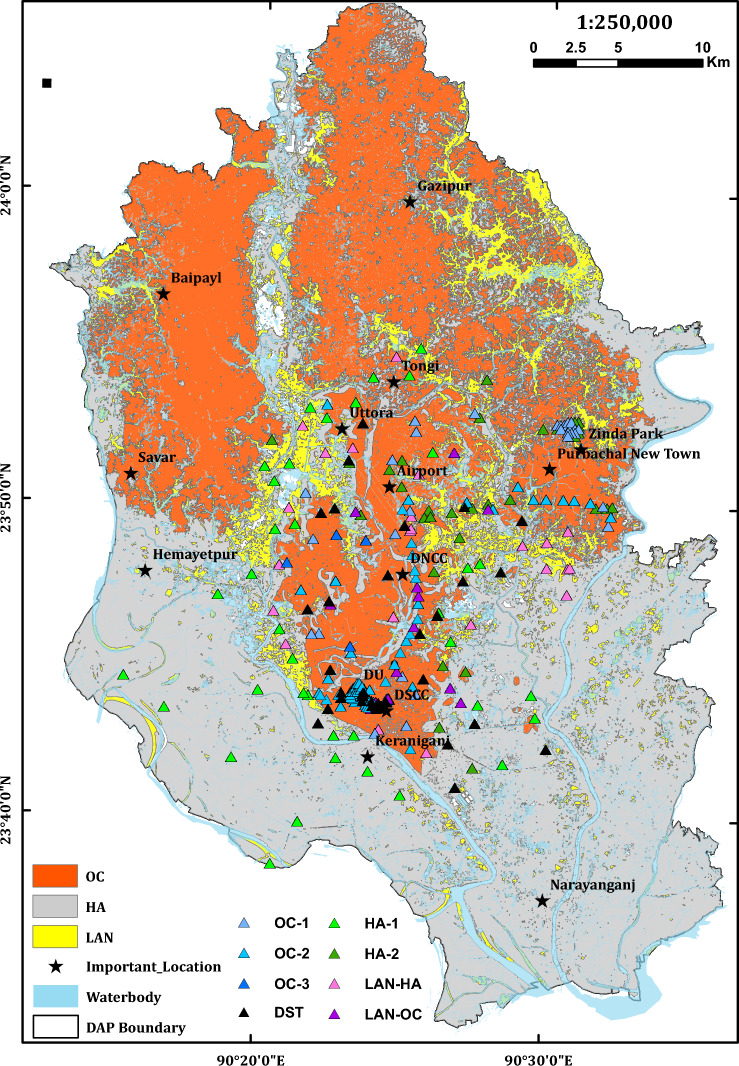
Simplified engineering geomorphic unit-based map of DAP of Dhaka City. OC: Pleistocene terrace (Overconsolidated clay/silty clay/clayey silt), HA: Holocene alluvium (Unconsolidated sand, silt and clay), LAN: Landfill (Mainly unconsildated sand). This map was produced from image analysis and verified with borehole data. (Sources of Borehole (BH) and Downhole Seismic Test (DST) data: Dhaka University (DU), Geological Survey of Bangladesh (GSB), Creative Soil Investigations, Prosoil, Comprehensive Disaster Management Programme (CDMP))

Shear wave velocity (Vs) of the near-surface soils is critical in seismic site response analysis^51,52^. Although it is costly and not simple like SPT, it has advantages over SPT-N value^53^. For example, measurements can be taken in hard soils where the SPT and Cone Penetration Test (CPT) are impossible or not permitted or in areas where undisturbed samples are difficult to collect. In addition, it is a fundamental mechanical property of soil. Therefore, it is directly related to the small-strain shear modulus, a required parameter in analytical procedures for estimating dynamic soil response, site response analysis, and soil-structure interaction analyses^54^. The study’s Downhole Seismic Test (DST) locations are shown in Fig. 2. The DST is a direct method to estimate the Vs. and is considered the most reliable and accurate^55^. Supplementary Fig. S3 displays the Vs—profile obtained through the downhole seismic test (PS logging).

However, where DST data are unavailable to estimate Vs., we have used the empirical equation proposed by Rahman et al.^35^ to estimate Vs from SPT-N value. The used equation for shear wave velocity is given below:

1 $$V_{S} = 92.1 \times N^{0.337} \left( {{\text{All soils}}} \right)$$

Finally, the average shear wave velocity of the top 30 m (Vs30) is calculated using the following equation:

2 $$V_{s } 30 = \frac{30}{{\mathop \sum \nolimits_{i = 1}^{N} h_{i} /v_{i} }}$$

where, ${h}_{i}$ and ${v}_{i}$ denote the thickness (m) and Vs (m/s) of the *i*th layer, respectively, and $N$ denotes the number of layers in the top 30 m depth.

The shear wave velocity profiles of all geomorphic units are given in Supplementary Fig. S4.

#### Shear modulus reduction and material damping curves

Shear modulus and soil material damping curves are prerequisites for nonlinear site response analysis^56^. As shear modulus and damping ratio reduction curves are not available for Dhaka city, it is a prevalent practice to use standard curves from the literature. The widely used curves for categorizing dynamic soil behavior are represented in many studies^57–59^. In this study, to fit the damping ratio and shear modulus reduction curve, as this empirical model is one of the most acceptable among practitioners for determining the variability of soft soil nonlinear properties, are used from Darendeli^57^. Darendeli^57^ modified the original hyperbolic model recommended by Hardin and Drnevich^18^. The Darendeli (2001) modified curve has five variables: effective vertical stress, over-consolidation ratio (OCR), number of loading cycles, plasticity index (PI), and loading frequency. The OCR measures from the empirical equation of

3 $${\text{OCR}} = \frac{{\sigma_{p}^{^{\prime}} }}{{\sigma_{yy}^{^{\prime}} }}$$

where, $\sigma_{p}^{^{\prime}}$: pre-consolidation stress and $\sigma_{yy}^{^{\prime}}$: natural pressure. If the ratio becomes more than one, the soil is over-consolidated; equal to one usually means consolidated, and less than one implies under-consolidation^60^. The PI of soils was collected from laboratory tests. The number of loading cycles in the paper used is ten recommended by the DEEPSOIL manuals. The effective vertical stress of the soil layer was calculated by multiplying the soil layer thickness with the unit weight of the soil. If the soil is saturated, then pore water pressure will be minus from the vertical stress. Then, the effective vertical stress becomes $\overline{\sigma } = \gamma *h - \gamma_{w} *h$; where h is thickness and γ: unit weight of soil, ${\gamma }_{w}:$ unit weight of water^61^. Using the soil PI, OCR, and the number of loading cycles, this model can predict the baseline of the damping ratios and the modulus reduction curves. Supplementary Fig. S5 illustrated the reference^57^, fit and current curves of shear modulus, damping ratio, and shear strength.

#### Strong ground motion selection

The strong ground motion time history (acceleration, velocity, and displacement) data is a prerequisite for dynamic response analysis of soil and structure. However, selecting strong ground motion is challenging as it depends not only on epicentral distance and rupture length but also on other factors, such as subsurface geology, directivity, etc. In the case of a moderate seismicity-prone and developing country, Bangladesh, the lack of strong ground motion data (Mw > 7.0 earthquake did not occur for more than 100 years in Bangladesh) is becoming more challenging. Therefore, we have used the most widely used spectral matching technique in this study to select the strong ground time history acceleration data. Firstly, we have downloaded strong motion raw data (horizontal component, H1) from the PEER NGA WEST2 database considering the similar response spectra to the Bangladesh National Building Code (BNBC) 2020 response spectra (target response spectra) for Maximum Credible Earthquake (MCE) and SB type soil condition (Vs 360 to 800 m/s; Supplementary Fig. S6)^36^. The details of the downloaded earthquake accelerograms are given in Supplementary Table S1. Afterward, we adjusted earthquake accelerograms by matching them with BNBC 2020 SB_MCE response spectra^36^ using the wavelets algorithm proposed by Atik and Abrahamson^41^. Note that we have used SeismoMatch for spectral matching. Supplementary Fig. S7 shows the matched and target response spectra (BNBC 2020 SB_MCE). In addition, for this study, we have generated two artificial earthquake accelerograms matched to target response spectra (BNBC 2020 SB_MCE) by using SeismoArtif software. Both far-field inter and intra-plate earthquakes have been considered to generate synthetic accelerograms. In summary, seven earthquake accelerograms from seven earthquakes and two synthetic accelerograms have been considered for site response analysis. Supplementary Fig. S8 illustrates the detailed (step-by-step) procedure of ground motion selection for the Kobe 1995 earthquake.

#### Computing one-dimensional surface site response analysis

When motion is propagated vertically and horizontally, 1D site response analysis evaluates the soil column's impacts on earthquake time histories vertically. Frequency domain and time-domain analysis are the most often used techniques in 1D analysis. However, time-domain response analysis is more realistic than frequency-domain analysis as it consider changes in soil characteristics over time and the propagation of input motion into the soil^62^. The nonlinear time domain site response analysis was performed based on two model backbone curves and the hysteresis behavior of soil in terms of shear stress–strain loading–unloading.

The dynamic response of multi-degree-of-freedom systems exposed to base excitation is determined by nonlinear site response. This study utilizes DEEPSOILv7 software^23^ for calculating nonlinear response analysis. Determining nonlinear, linear, and equivalent linear response analysis in DEEPSOIL is possible. Several soil constitutive models are accessible in DEEPSOIL, including the MKZ model with pressure-dependent behavior^63^ and the new General Quadratic/Hyperbolic (GQ/H) model with non-masing requirements^64^. Numerous models for reference fit curves for shear modulus reduction and damping ratio curves are also available in this computer program. Among all the models, the GQ/H model is used as it is updated, widely used, and has the best fit for sandy and clayey soils. The GQ/H model for nonlinear site response analysis is based on the physics of wave propagation in heterogeneous media. The GQ/H model assumes that a quadratic-hyperbolic function can represent the stress–strain relationship of the soil. This quadric model used two lines of initial shear strength and shear strength at failures that capture small and large strain behavior of soil into a continuous curve because within linear boundaries initial shear strength and shear strength at failure intersect at some reference shear strain and create backbone curve^64,65^. The equation for the backbone curve of the GQ/H model is given below:

4 $$\frac{\tau }{{\tau }_{max}}=\frac{2(\frac{\gamma }{{\gamma }_{r}})}{1+\left(\frac{\gamma }{{\gamma }_{r}}\right)+\sqrt{{\{1+\left(\frac{\gamma }{{\gamma }_{r}}\right)\}}^{2}-4{\theta }_{r}(\frac{\gamma }{{\gamma }_{r}})}}$$

$\tau$ is the shear stress, ${\tau }_{max}$ is the shear strength at failure, γ is the shear strain, ${\gamma }_{r}$ is the reference shear strain and ${\theta }_{r}$ is a curve fitting parameter used to adjust the curves of shear stress and strain curves and has no effect on boundary conditions. The shear strength at failure was measured using Mohr–Coulomb failure Criteria. Mohr–Coulomb failure criteria is given below

5 $$\tau_{max} = {\text{c}} + \sigma^{^{\prime\prime}} *{\text{tan}}\varphi$$

Here, ${\tau }_{max}$ is the shear strength of soil, c is the cohesion of soil, $\sigma^{^{\prime\prime}}$ is effective stress, and φ is the frictional angle of soil. The cohesion and frictional angle are measured by lab test and given in Table 1. The effective stress measurement is already mentioned above. From Eq. (5), estimated shear strength was used as a user-defined parameter in DEEPSOIL for the GQ/H model^66^. The backbone curve uses Konder^67^ proposed values as the reference shear strain^64,65^. The GQ/H model attempts to become flexible with minor strain soil behavior while simultaneously introducing shear strength failure. After the backbone curve created from the GQ/H model, the curve incorporates with hysteresis behavior of soil. In the hysteresis model, the shear stress–strain curve follows the backbone curve as τ = ${F}_{bb}(\gamma )$ for initial shear loading. Therefore, the backbone curve of GQ/H model becomes

6 $$\tau ={\tau }_{max}\left[\frac{2\left(\frac{\gamma }{{\gamma }_{r}}\right)}{1+\left(\frac{\gamma }{{\gamma }_{r}}\right)+\sqrt{{\left\{1+\left(\frac{\gamma }{{\gamma }_{r}}\right)\right\}}^{2}-4{\theta }_{r}\left(\frac{\gamma }{{\gamma }_{r}}\right)}}\right]={F}_{bb}(\gamma )$$

The nonlinear time domain site response uses the extended unloading -loading masing rules to model hysteresis behavior. However, using masing rules in hysteresis damping calculation can overestimate damping in large strain. Therefore, Philips and Hashash^68^ proposed a modulus reduction and damping factor (MRDF) approach. When this MRDF approach is used with the GQ/H model, the damping reduction matches well with the reference curves of Darendeli^65^. The MRDF reduction factor is

7 $${\text{F}}(\gamma_{max} ) = p_{1} - p_{2} \left( {1 - \frac{{G_{{\gamma_{max} }} }}{{G_{max} }}} \right)^{{p_{3} }}$$

where, ${p}_{1}, {p}_{2}$ and ${p}_{3}$ are nondimensional parameters selected to obtain the best fit with the target damping curve. After defining the soil model parameters, the reduction factor parameters are chosen to best fit the damping curve. The computer program already has a built-in correlation of the GQ/H model and MRDF parameters for the best-fitted damping curve^65^. The MDRF model was developed for non-masing rules in the cyclic behavior of soil^69^. As observed in laboratory tests, the damping behavior of soils can be better captured by a non-Masing hysteresis model that shrinks the size of hysteresis loops. The used non-Masing hysteresis model considers that the soil's response may change over time as it undergoes deformation and stress. This model can capture the damping behavior more accurately by shrinking the size of the hysteresis loop, which represents the difference in energy input and output over one loading cycle.

#### Computing amplification factor (AF)

Site coefficient or Amplification Factor (AF) is commonly used to express ground motion amplification at different spectral periods. We have used Eq. (8) to estimate the AF at different spectral periods.

8 $$\mathrm{A}\left(\mathrm{T}\right)= {\mathrm{R}}_{\mathrm{soil }}(\mathrm{T})/{\mathrm{R}}_{\mathrm{input motion }}(\mathrm{T})$$

where A is the amplification value of T second, ${\mathrm{R}}_{\mathrm{soil}}$ is the response of soil on the surface at T second, and ${\mathrm{R}}_{\mathrm{input motion}}$ is the input motion of earthquake at T second^70,71^.

## Results

### Engineering geomorphic map

The engineering geomorphic unit map of the DAP of the Dhaka City area was prepared and verified with the boreholes and surface geological data. The region has been divided into three simplified geomorphic units. They are the Holocene deposit (HA), Pleistocene deposit (OC), and Landfill (LAN) (Fig. 2). Nearly 50% of the area comprises Holocene deposits, and 33% includes Pleistocene deposits. The sticky, stiff to very stiff silty clay or clayey silt and reddish brown to yellowish brown color is the unique characteristics of OC underlain by medium dense to dense silty sand^34^. HA is mainly composed of light grey to dark grey color, very soft to soft clayey silt or silty clay, and gray to brown, loose to dense sandy silt or silty sand^34^. The common mineral components of the HA unit are quartz, feldspar, and mica^35,37,72^. The LAN unit is characterized by grey loose silty sand to sand and soft clayey silt. River dredging sand-filling sites are very vulnerable to liquefaction hazards; for example, the 1995 Kobe earthquake caused massive liquefaction in Port Island's reclaimed soil deposits.

### Engineering geomorphic unit-wise soil properties

**Figure 3:**
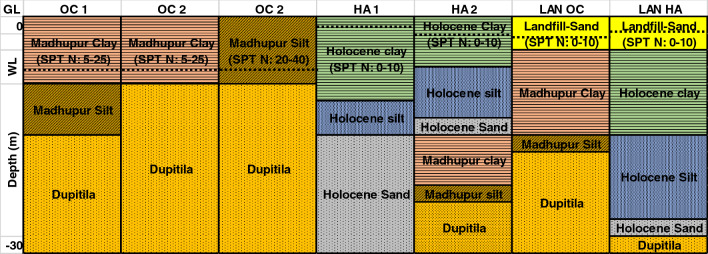
Soil profiles of OC, HA, LAN. The soil profiles of identified seven geomorphic sub-units of Dhaka city DAP are shown with each layer's average thickness. The OC soils consist of Madhapur clay, Madhapur silt, and Dupitila sand. The HA consists of Holocene clay, Holocene silt, and Holocene sand in some areas underlain by OC 1. The LAN unit with an average of 3 m filling materials underlain by HA or OC. The figure was prepared using Microsoft Excel.

We have identified seven types of soil profiles for the study area's major categorized geomorphic units. HA deposits cover most of the DAP region, followed by OC (Fig. 2). Among the seven types of soil profiles (geomorphic sub-units), two types included HA, two types in LAN, and three classes in OC (Fig. 3).

As mentioned, we have identified three prominent varieties of OC geomorphic units: OC 1, OC 2, and OC 3. OC 1 consists of three primary soil layers: Madhupur clay, Madhupur silt, and Dupitila formation (Fig. 3). OC 2 consists of Madhupur clay underlain by Dupitila formation, and OC 3 consists of Madhupur silt and Dupitila formation (Fig. 3). The identified two prominent verities of HA are HA 1 and HA 2. HA 1 consists of, from top to bottom, Holocene clay, Holocene silt, and Holocene sand. However, HA 2 consists of HA 1 and OC 1, where OC 1 is overlain by HA 1 (Fig. 3). In addition, there are two types of LAN soil units where LAN overlies OC (LAN OC) and LAN underlain by HA (LAN HA). The average filling depth in these two types is 3–4 m, and most of the LAN was composed of loose, unconsolidated river sand. Landfill sand and Holocene sand are susceptible to liquefaction. The liquefaction phenomenon will appear on the surface or not; it depends on the relative thickness of the liquefiable and non-liquefiable layer ^73^. In addition, the Liquefaction Potential Index (LPI) value is also a good and widely used criterion for estimating surface manifestations of liquefaction hazards^74^. Engineering properties of the identified seven soil profiles of three geomorphic units are given in Table 1.

**Table 1:** Engineering properties (Average) of pleistocene deposit (overconsolidated clay (OC)), holocene deposit (HA), and landfill (LAN). The engineering soil properties of all seven sub-units are presented in this table. The values of properties are shown in ranges.

Unit Name	Layer type	% of sand	% of Fine	Thickness (m)	SPT N value range	Unit Weight (kN/m3)	Vs. (m/s)	C (kN/m2)	Friction ($^\circ$)	PI	Density (gm/cc)	Vs30 (m/s)
OC 1	Madhupur clay	< 10	> 90	≈ 7.5	5–25	≈ 19	200–250	50–200	< 10	25–35	≈ 2.00	220–350
	Madhupur silt	< 20	> 80	≈ 6	20–40	≈ 18	220–300	100–300	< 15	25–35	≈ 1.9	
	Dupitila	> 60	< 40	≈ 17	30–60	≈ 20	300–400	< 15	30–40	–	≈ 2.1	
OC 2	Madhupur clay	< 10	> 90	≈ 8	5–25	≈ 19	200–250	50–200	< 10	25–35	≈ 2.0	250–350
	Dupitila	> 60	< 40	≈ 22	30–60	≈ 20	300–400	< 10	35–40	–	≈ 2.1	
OC 3	Madhupur silt	< 10	> 90	≈ 7	20–40	≈ 18	220–300	100–300	< 15	25–35	≈ 1.9	250–350
	Dupitila	> 60	< 40	≈ 23	30–60	≈ 20	300–400	< 10	35–40	–	≈ 2.1	
HA 1	Holocene clay	< 10	> 90	≈ 12	0–10	≈ 16	120–180	40–100	< 10	20–25	≈ 1.6	150–180
	Holocene silt	< 20	> 80	≈ 7	10–25	≈ 17	150–200	20–50	< 20	15–25	≈ 1.7	
	Holocene sand	> 60	< 40	≈ 11	20–35	≈ 18	180–250	< 20	20–30	5–12	≈ 1.8	
HA 2	Holocene clay	< 10	> 90	≈ 7.5	0–10	≈ 16	120–180	40–100	< 10	20–25	≈ 1.6	180–250
	Holocene silt	< 20	> 80	≈ 4.5	10–25	≈ 17	150–200	20–50	< 20	15–25	≈ 1.7	
	Holocene sand	> 60	< 40	≈ 2	20–35	≈ 18	180–250	< 20	20–30	5–12	≈ 1.8	
	Madhupur clay	< 10	> 90	≈ 5	5–25	≈ 17	200–250	50–200	< 10	20–35	≈ 2.00	
	Madhupur silt	< 20	> 80	≈ 2	20–40	≈ 18	220–300	100–300	< 15	25–35	≈ 1.9	
	Dupitila	> 60	< 40	≈ 9	30–60	≈ 19	300–400	< 15	30–40	–	≈ 2.0	
LAN-OC	Landfill	> 90	< 10	≈ 3	0–10	≈ 16	120–180	< 10	25–30	–	≈ 1.7	200–300
	Madhupur Clay	< 10	> 90	≈ 7.5	5–25	≈ 19	250–300	100–200	< 10	25–35	≈ 2.00	
	Madhupur Silt	< 20	> 80	≈ 1.5	20–40	≈ 18	250–300	100–300	< 15	25–35	≈ 1.9	
	Dupitila	> 60	< 40	≈ 18	30–60	≈ 19	300–400	< 15	30–40	–	≈ 2.0	
LAN-HA	Landfill	> 90	< 10	≈ 3	0–10	≈ 17	120–180	< 10	< 20	–	≈ 1.8	150–200
	Holocene Clay	< 10	> 90	≈ 9	0–10	≈ 16	120–180	40–100	< 10	20–25	≈ 1.6	
	Holocene Silt	< 20	> 80	≈ 10	10–25	≈ 17	150–200	20–50	< 20	15–25	≈ 1.7	
	Holocene Sand	> 60	< 40	≈ 2	20–35	≈ 18	180–250	< 20	20–30	5–12	≈ 1.8	
	Dupitila	> 60	< 40	≈ 5	30–60	≈ 19	300–400	< 15	30–40	–	≈ 2.0	

### Site response analysis

**Figure 4:**
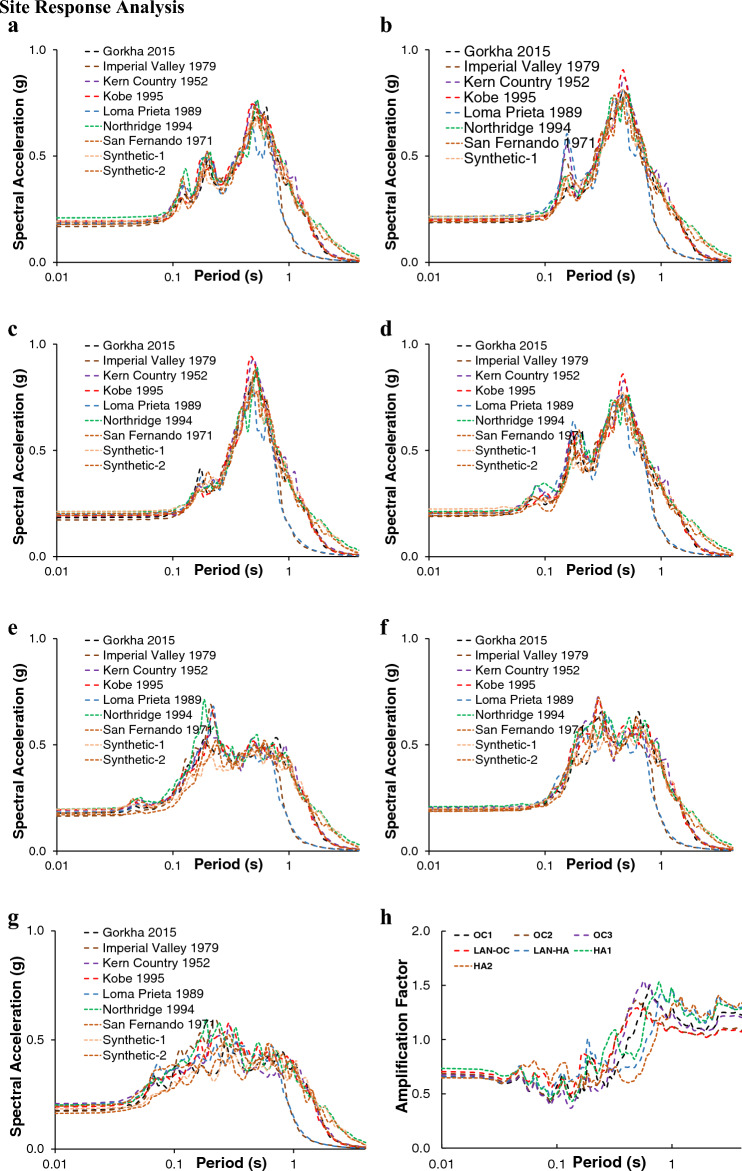
All graphs exhibit the ground surface's nonlinear response spectrum of the seven geomorphic subunits (**a**) Response spectra of OC 1; (**b**) Response spectra of OC 2; (**c**) Response spectra of OC 3; (**d**) Response spectra of LAN-OC; (**e**) Response spectra of LAN-HA; (**f**) Response spectra of HA 1; (**g**) Response spectra of HA 2 for selected earthquakes' strong motion input; (**h**) AF for the Gorkha earthquake 2015 input motion. The response analysis is performed in DEEPSOIL software (http://deepsoil.cee.illinois.edu/), and figures are drawn in Excel.

The geomorphic unit-based nonlinear surface response spectra for nine selected input ground motions are illustrated in Fig. 4. The response spectrums indicate various outcomes depending on the geomorphic unit and input earthquakes ground motion; however, the overall observation is that spectral acceleration is de-amplified (ground motion amplitude decreases compared to input ground motion) in the short period and amplified (amplitude of ground motion increase compared to input ground motion) in the long period. All the responses are measured considering a 30 m soil column where Vs is 360–450 m/s at the bottom of the soil column.

As we know, earthquake ground motion on the ground surface depends on the source, path, and soil characteristics of selected sites. The ground motion with definite frequency interacts with the predominant frequency of soil; as a result, a variation in the response spectrum on the surface is observed. Figure 4, represents the response spectral acceleration of OC, HA, and LAN units. The OC unit has three subunits namely: OC 1, OC 2, and OC3. Figure 4a–c represent the response of OC 1, OC 2, and OC3, respectively. The OC soil (OC 1, OC 2, and OC 3) (Fig. 4a–c) represents that the responses are de-amplified in a short period (0.1 s) while peak acceleration at around 0.7 s which means the response is amplified in the long period. However, similar responses are observed from OC 1, OC 2, and OC 3, as the Madhupur Clay and Silt engineering properties are close (Table 1). Figure 4d,e show the response spectrum for LAN-HA and LAN-OC, respectively. As expected, responses from LAN units (LAN-HA and LAN-OC) are not identical. The LAN-HA shows predominant peak acceleration at 0.2 s (Fig. 4d), whereas the LAN-OC unit show peak response at around 0.5 s (Fig. 4e). The upper 3 m of the LAN unit is filled with loose river sand (Table 1). After the 3 m depth, the LAN-OC unit has Madupur clay, silt, and Dupitila layer and LAN-HA has Holocene clay, silt, sand, and Dupitila formation. This means that two LAN units distinguish soil types and properties. These different soil properties and layer thicknesses cause different responses for LAN units. The last unit is the HA unit, HA 1 and HA 2. HA 1 means the soil up to 30 m entirely consists of Holocene clay, silt, and sand, whereas HA 2 consists of both Holocene clay, silt, sand, Madupur clay, silt, and Dupitila sand. The spectral acceleration of HA 1 and HA 2 illustrated in Fig. 4f,g, respectively. Figure 4f,g show slightly different response spectra than other units. The predominant period of the considered soil column ranges from approximately 0.40 s to 0.50 s, and the input ground motion predominant period is mainly concentrated around 0.40 s (from 0.22 to 0.48 s), expected Loma Prieta (around 0.22 s). Therefore, although Loma Prieta has the highest matched PGA (0.31 g), its response is less. On the other hand, Kobe (peak period 0.46 s) has matched PGA of 0.27 g and shows a higher response than Loma Prieta. The predominant period of OC 1, OC 2, OC 3, and LAN-OC is around 0.40 s; therefore, their responses are similar as expected. On the other hand, the predominant period of HA 1, HA 2, and LAN-HA is around 0.50 s, showing similar responses. This analysis calculates response spectra concerning frequency-independent viscous damping ratio using MRDF curve fitting for Darendali's curve fitting factor^68^. Figure 4h illustrates the AF of seven geomorphic subunits at different spectral periods for Gorkha earthquake input. The AF is decreased (< 1) in short period (before approximately 0.5 s) and increased (> 1) in a long period (after approximately 0.5 s).

**Figure 5:**
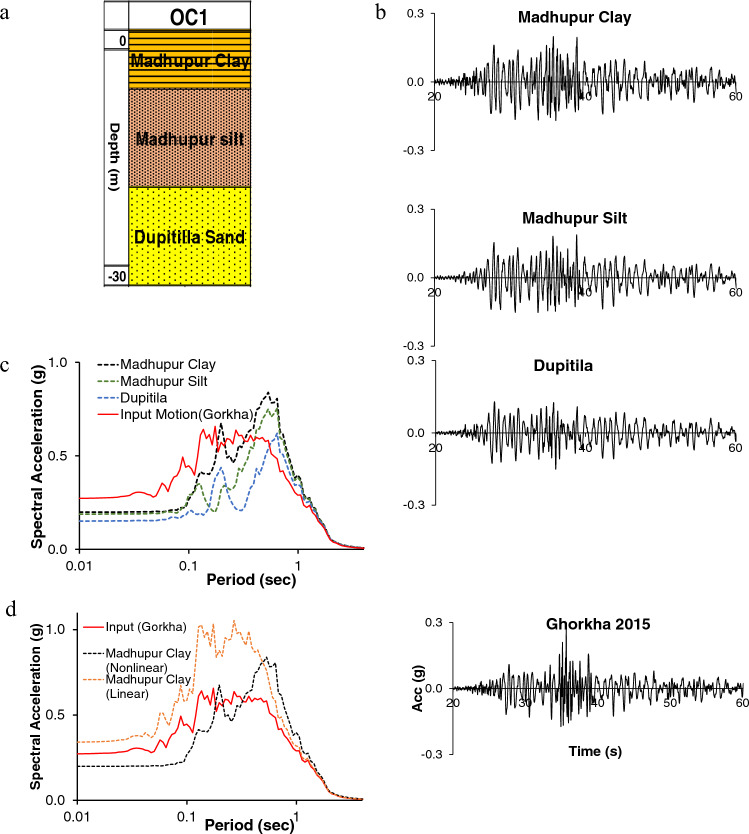
The layers-wise nonlinear response for OC 1. (**a**) Soil profile of OC 1, (**b**) Layer-wised nonlinear response of OC 1 and input ground motion (Gorkha 2015 earthquake), (**c**) Nonlinear response spectrum for OC1, and (**d**) Spectral acceleration for OC 1 (Madhupur clay) in terms of linear and nonlinear analysis. The figures are drawn in Excel and response calculations are performed in DEEPSOIL.

The layer-wise nonlinear response for OC 1 unit is illustrated in Fig. 5. As expected, the top layer, Madhupur clay, has a maximum response spectrum than Madhupur silt and Dupitila Formation (Fig. 5c) compared with the input earthquake motion (Gorkha 2015), the input motion has maximum spectral acceleration at 0.17 s with 0.66 g. In contrast, the responses have maximum spectral acceleration at 0.53 s with 0.83 g (Fig. 5c). The responses are de-amplified in a short period until 0.1 s. Figure 5d shows the difference between the linear and nonlinear responses of Madhupur clay. Spectral acceleration from the linear analysis is overestimated in the short period and underestimated in the long period. However, the nonlinear response shows de-amplification in a short period and amplification in a long period. As most of the soil of the DAP area is relatively soft, the response values would be overestimated using linear analysis because the linear model cannot effectively predict ground motion during the short period when peak shear strain is between 0.01 and 0.1% ^13^.

## Discussion

In this study, site responses are estimated for seven types of engineering geomorphic sub-units under three main units using nonlinear analysis. We observed that the response spectrum shifted towards the long period with maximum acceleration more significant than the input ground motion (Figs. 4 and 5). The soft/loose sandy (LAN (mainly loose sand) (SPT N value 0–10)) and clayey (HA (SPT N value 0–10) and Madhupur clay (SPT N value 5–25)) soil up to 30 m depth is responsible for amplifying acceleration over a long period and de-amplification of acceleration in short periods. However, according to the National Earthquake Hazard Reduction Program (NEHRP) site AF, for 30 m soil depth, the response is amplified in a short period^52^. On the other hand, the soft/loose sandy and clayey soil of DAP of Dhaka city area does not follow the NHERP site AF and shows de-amplification over a short period (Fig. 4h).

In a nonlinear analysis, the higher frequencies are amplified at high shear moduli, whereas the lower frequencies are amplified at low shear moduli (Supplementary Fig. S5). The de-amplification and amplification on the response spectrum depend on the shear wave velocity and the other soil properties. Hashash and Park^75^ showed that because of the decreased shear wave velocity at the top 70 m of the profile, the HA profile exhibits less amplified acceleration in short periods than the Pleistocene profile and a slight shifting to longer periods.

De-amplification or amplification of the ground surface response spectrum mainly depends on input ground motion characteristics and soil properties (loose or dense, soft or stiff). The soil shows a response according to the frequency and amplitude of the ground motion. As we know, soft soil damps high-frequency ground motion and amplifies low-frequency ground motion. In this study, due to the presence of nonlinear soft clay, silt, and very loose sand deposits, the short-period ground motion de-amplified, and the long-period ground motion amplified. When the input motion frequency matches the soil's natural frequency, it amplifies the input motion.

The HA covers most of the study area. OC and an artificial LAN cover the rest of the site. The response spectrums are different according to soil type and shear wave velocity. We can see that OC with maximum acceleration over a long period than HA and LAN deposits. On the contrary, HA exhibits long-lasting amplification than OC. The artificial LAN shows a mixture of them. High frequencies are attenuated more than low frequencies when passing through a column of soft, loose soil that consists primarily of sandy soil. High frequencies are amplified more in OC sediments than in thick and soft HA due to the higher natural frequencies of OC sediments. Thicker Holocene sediments have lower natural frequencies, resulting in amplification in the lower frequencies than the OC sediments. Dhaka City is very susceptible to far-field earthquakes. The low-frequency tremor with adequate amplitude can cause significant damage to low-frequency structures due to resonance effects.

In summary, for the considered earthquakes, the near-surface soil response of DAP of Dhaka city shows de-amplification of acceleration in the short period and amplified acceleration in the long period. This type of spectrum is controlled by the characteristics and properties of soft clayey and sandy soil, and response spectra differ according to the geomorphic unit. For example, the Holocene soil unit accelerates toward a long period more than the Pleistocene unit. However, the Pleistocene deposits exhibit higher acceleration values than those of the Holocene.

## Conclusion

This paper examined the engineering geomorphic unit-based nonlinear site response of the DAP area of Dhaka City. First, the simplified engineering geomorphic unit map was prepared by image analysis and then verified using borehole data and the surface geology map of GSB. The identified three major geomorphic units are OC, HA, and LAN. Further, OC, HA, and LAN are divided into OC 1, OC 2, OC 3, HA 1, HA 2, LAN-OC, and LAN-HA sub-units considering the subsurface soil layer.

In total, nine input ground motions (seven from PEER NGA WEST2 data set and two synthetics) were used for 1D nonlinear site response analysis of seven geomorphic sub-units of DAP of Dhaka city. The overall observation from the nonlinear soil site response analysis is that the responses are de-amplified in the short period while amplified in the long period.

OC's spectral acceleration amplitude is higher than HA units. However, HA units show longer peak spectral acceleration duration than OC units. This phenomenon might cause the resonance of tall buildings on HA units, consequently increasing the probability of damage. As expected, LAN-OC units show similar behavior of OC and LAN-HA to HA. Dhaka city's vigorous vertical and lateral expansion has caused it to create a large built environment over soft soil. Therefore, the findings of this study can be used as an essential indicator to prepare a seismic risk-sensitive land use plan for the future development of the DAP of Dhaka City to reduce the seismic risk. A similar procedure could be adopted for other earthquake-vulnerable cities to understand the seismic risk.

## Supplementary Information

Supplementary Information.

## Data Availability

Strong Ground Motion Data are available at https://ngawest2.berkeley.edu/. The SPT and DST data from mentioned organizations could be appropriately acquired by contact with them.

## Code availability

Data analysis is performed using Excel, DEEPSOIL, AgrcGIS and Seismosoft.
