# Supplementary material for: Simplified engineering geomorphic unit-based seismic site characterization of the detailed area plan of Dhaka city, Bangladesh
Source: Sci Rep. 2023 Jul 10;13:11151. doi: 10.1038/s41598-023-37628-6 (PMC10333223; doi:10.1038/s41598-023-37628-6)
Supplement: Supplementary file 1 — Supplementary Information. [file 41598_2023_37628_MOESM1_ESM.docx]

**Simplified Engineering Geomorphic Unit-based Seismic Site Characterization of the Detailed Area Plan of Dhaka City, Bangladesh**

Md Shakhawat Hossain^1, 2*^, Muneyoshi Numada^1*^, Momtahina Mitu^3^, Kishor Timsina^1^, Chaitaniya Krisna^4^, Md. Zillur Rahman^2^, A.S.M. Maksud Kamal^2^, Kimiro Meguro^1^

^1^Institute of Industrial Science, the University of Tokyo, Tokyo, Japan

^2^Department of Disaster Science and Climate Resilience, University of Dhaka, Dhaka, Bangladesh

^3^Department of Coastal Studies and Disaster Management, University of Barisal, Barisal, Bangladesh

^4^Department of Civil and Infrastructure Engineering, Asian Institute of Technology, Thailand

*^*^Corresponding authors' email:*

[*shakhawat.dsm@du.ac.bd;*](mailto:shakhawat.dsm@du.ac.bd;%20%20)

[*numa@iis.u-tokyo.ac.jp*](mailto:numa@iis.u-tokyo.ac.jp)

**Supplementary information**

**Supplementary Figures**


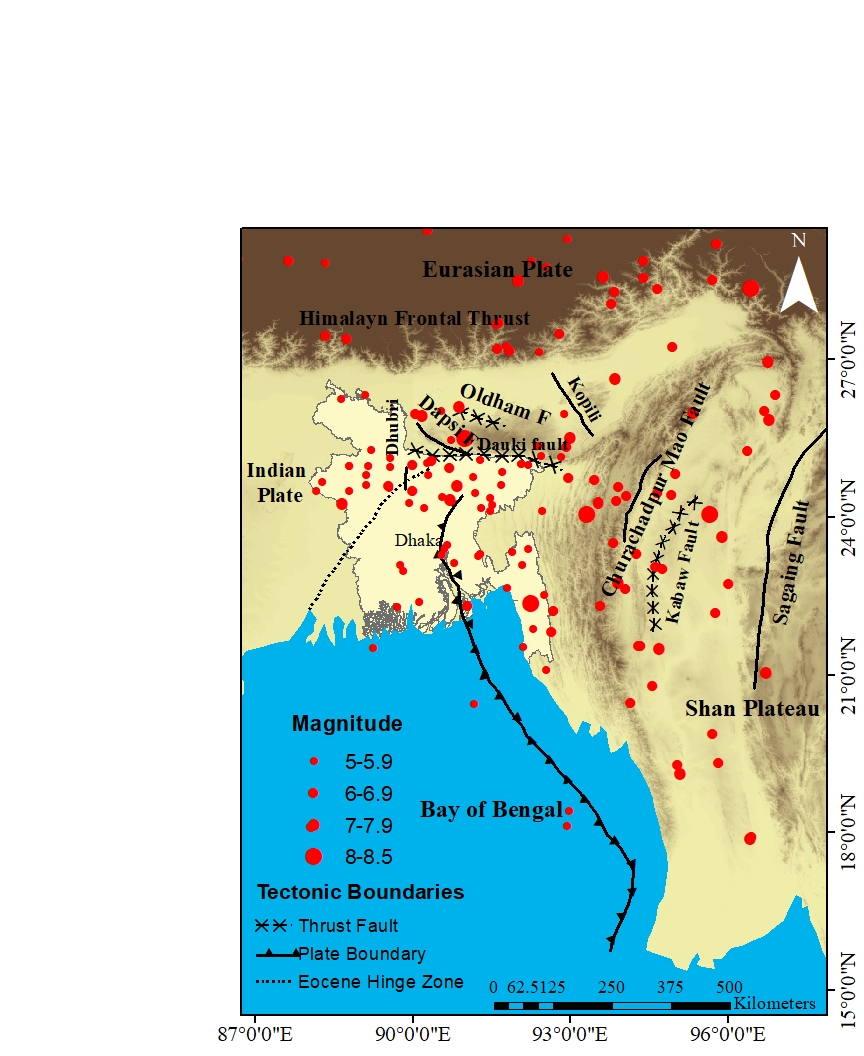


**Supplementary Figure S1**: Map showing historical earthquakes from 1762 to 2020.

| 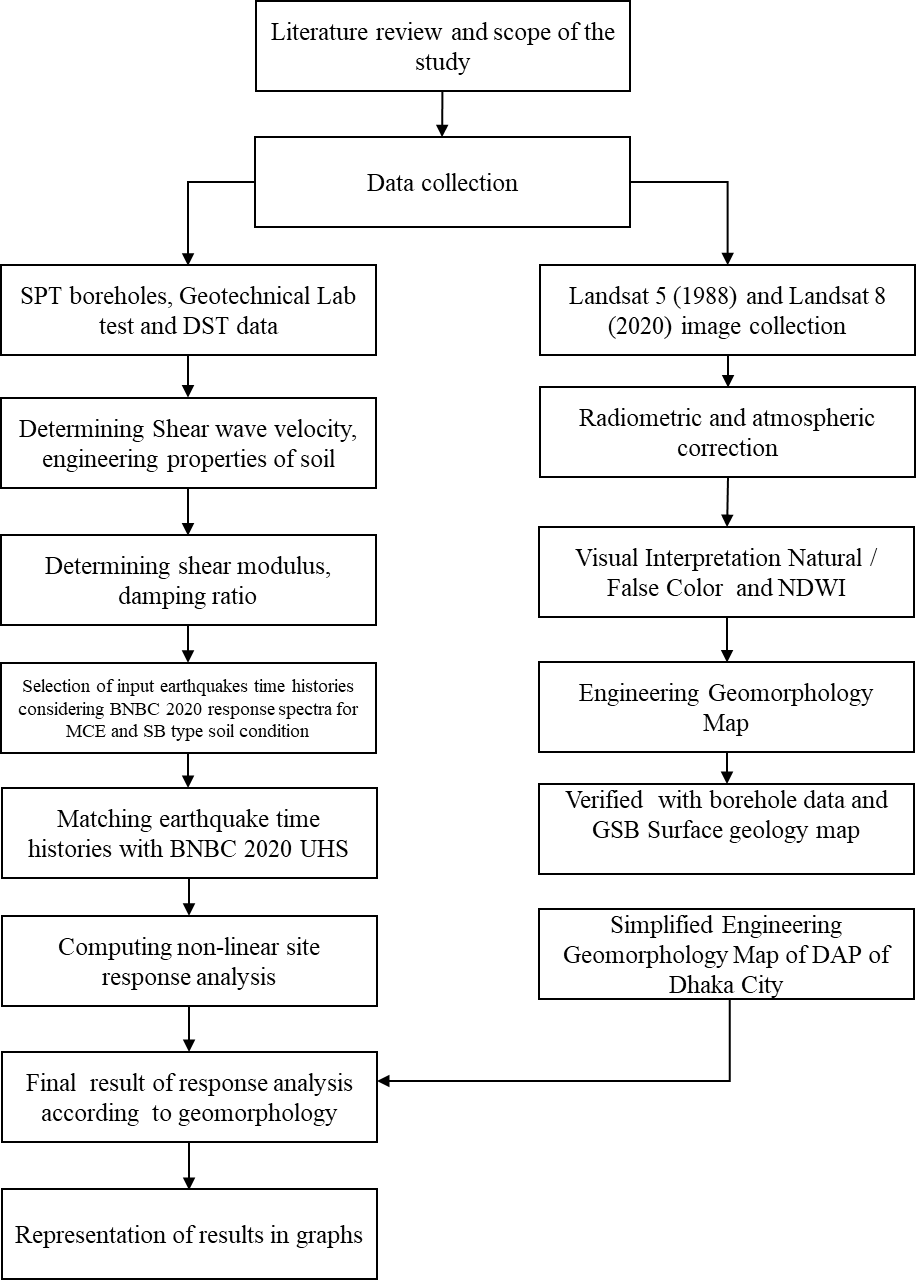 |
| --- |

**Supplementary Figure S2**: Methodological framework

| (a) Time distance curve | (b) Layer velocity |
| --- | --- |
|  |  |
| **Supplementary Figure S3**: (a) Time distance curve and (b) layer velocity obtained from the downhole seismic test | |

|  | **Supplementary Figure S4**: Average Shear wave velocity profile of geomorphic sub-units up to 30m depth |
| --- | --- |

|  |  |
| --- | --- |
|  | **Supplementary Figure S5**: Example of current and fit curves of shear modulus reduction curve and damping ratio for clayey soil (depth: 7.5 m, plasticity index: 29, over-consolidation ratio: 2) with reference curve. |

|  |
| --- |
| **Supplementary Figure S6**: Pre-match response spectra with target response spectra (BNBC 2020 SB_MCE) |
|  |
| **Supplementary Figure S7**: Matched response spectra with target response spectra (BNBC 2020 SB_MCE) |

|  |
| --- |
| (a) Initial (Pre-match) acceleration time history of Kobe 1995 earthquake (Step-1) |
|  |
| (b) Spectral matching (Step 2) |
|  |
| (c) Frequency Distribution (Step-3) |
|  |
| (d) Arias Intensity (Step-4) |
|  |
| (e) Matched acceleration time history of Kobe 1995 earthquake (Step-5) |
| **Supplementary Figure S8**: Steps of Ground motion selection for Kobe 1995 earthquake. |

**Supplementary Figure S1**: Map showing historical earthquakes from 1762 to 2020 and the surrounding seismotectonic setup of Bangladesh. The earthquake data are collected from literature review^1,2^ and USGS. In addition, the plate boundary and background DEM map were collected from referenced paper^3^.

**Supplementary Figure S3**: Time distance and shear wave velocity of layer form downhole seismic test. These data are collected during field observation. (a) Time distance curve and (b) layer velocity obtained from the downhole seismic test**.**

**Supplementary Figure S4**: Average Shear wave velocity profile of geomorphic sub-units up to 30m depth. The shear wave velocities of all the geomorphic units were estimated from the downhole seismic test and empirical equation from SPT N values.

**Supplementary Figure S5**: Example of current and fit curves of shear modulus reduction curve and damping ratio for clayey soil. The shear modulus reduction curve and damping ratio for clayey and other soil layers are estimated from soil engineering properties. The figure shows three types of curves for clayey soil for 7.5m thickness, where over consolidation ratio is 2 and Plasticity Index is 29.

**Supplementary Figure S6**: Pre-match response spectra with target response spectra (BNBC 2020 SB_MCE). The original response spectra of the selected earthquakes are shown together. The response spectra of the selected historical earthquakes are collected from [Pacific Earthquake Engineering Research Center](https://peer.berkeley.edu/) website (<https://peer.berkeley.edu/peer-strong-ground-motion-databases>).

**Supplementary Figure S7**: Matched response spectra with target response spectra (BNBC 2020 SB_MCE. The collected earthquake time histories from PEER are matched with BNBC 2020SB_MCE. The matching is performed in SeismoMatch software (<https://seismosoft.com/products/seismomatch/>).

**Supplementary Figure S8**: Ground motion selection for Kobe 1995 earthquake. All the figures show the step of selecting ground motion. As an example, the selection of Kobe 1995 is represented here. **a**. Initial (Pre-match) acceleration time history of Kobe 1995 earthquake (Step-1), **b**. Spectral matching (Step-2), **c**. Frequency Distribution (Step-3), **d**. Arias Intensity (Step-4), and **e**. Matched acceleration time history of Kobe 1995 earthquake (Step-5)

**Supplementary Table**

**Supplementary Table S1**: List of selected different earthquakes' strong ground motion accelerations time history. (Source: PEER NGA WEST 2 and *NSET)

| Earthquake Name | Year | Station Name | Magnitude | Mechanism | Vs30 (m/sec) | Scale Factor | Component |
| --- | --- | --- | --- | --- | --- | --- | --- |
| "Kern County" | 1952 | Taft Lincoln School | 7.3 | Reverse | 385.43 | 1 | H1 |
| "San Fernando" | 1971 | LA - Hollywood Stor FF | 6.6 | Reverse | 316.46 | 1 | H1 |
| "Imperial Valley-06" | 1979 | Parachute Test Site | 6.4 | Strike slip | 348.69 | 1 | H1 |
| "Loma Prieta" | 1989 | Saratoga - Aloha Ave | 6.9 | Reverse Oblique | 380.89 | 1 | H1 |
| "Northridge-01" | 1994 | LA - UCLA Grounds | 6.7 | Reverse | 398.42 | 1 | H1 |
| "Kobe_ Japan" | 1995 | Tadoka | 6.9 | Strike-slip | 312 | 1 | H1 |
| *Gorkha | 2015 | Tribuvan | 7.8 | Thrust |  | 1 | EW |
| Synthetic-1 | Intra-plate Region, 100 Km, Mw 8 | | | | | | |
| Synthetic-2 | Inter-plate Region, 250 Km, Mw 8 | | | | | | |

**Supplementary Table S1**: List of selected different earthquakes strong ground motion accelerations time history. (Source: PEER NGA WEST 2 and *NSET)

**References**

1. Ambraseys, N. N. & Douglas, J. Magnitude calibration of north Indian earthquakes. *Geophys. J. Int.* **159**, 165–206 (2004).

2. Szeliga, W., Hough, S., Martin, S. & Bilham, R. Intensity, magnitude, location, and attenuation in India for felt earthquakes since 1762. *Bull. Seismol. Soc. Am.* **100**, 570–584 (2010).

3. Kamal, A. S. M. M., Mitu, M., Hossain, M. S., Rahman, M. M. & Rahman, M. Z. Seismic Hazard Analysis for the South-Central Coastal Region of Bangladesh Considering the Worst-Case Scenario. *Pure Appl. Geophys.* **178**, 2821–2838 (2021).
